# Accurate reporting of adherence to inhaled therapies in adults with cystic fibrosis: methods to calculate “normative adherence”

**DOI:** 10.2147/PPA.S105530

**Published:** 2016-05-23

**Authors:** Zhe Hui Hoo, Rachael Curley, Michael J Campbell, Stephen J Walters, Daniel Hind, Martin J Wildman

**Affiliations:** 1 School of Health and Related Research (ScHARR), University of Sheffield, Sheffield, UK; 2 Sheffield Adult Cystic Fibrosis Centre, Northern General Hospital, University of Sheffield, Sheffield, UK; 3 Sheffield Clinical Trials Research Unit, University of Sheffield, Sheffield, UK

**Keywords:** cystic fibrosis, medication adherence, nebulizers and vaporizers, epidemiologic methods

## Abstract

**Background:**

Preventative inhaled treatments in cystic fibrosis will only be effective in maintaining lung health if used appropriately. An accurate adherence index should therefore reflect treatment effectiveness, but the standard method of reporting adherence, that is, as a percentage of the agreed regimen between clinicians and people with cystic fibrosis, does not account for the appropriateness of the treatment regimen. We describe two different indices of inhaled therapy adherence for adults with cystic fibrosis which take into account effectiveness, that is, “simple” and “sophisticated” normative adherence.

**Methods to calculate normative adherence:**

Denominator adjustment involves fixing a minimum appropriate value based on the recommended therapy given a person’s characteristics. For simple normative adherence, the denominator is determined by the person’s *Pseudomonas* status. For sophisticated normative adherence, the denominator is determined by the person’s *Pseudomonas* status and history of pulmonary exacerbations over the previous year. Numerator adjustment involves capping the daily maximum inhaled therapy use at 100% so that medication overuse does not artificially inflate the adherence level.

**Three illustrative cases:**

Case A is an example of inhaled therapy under prescription based on *Pseudomonas* status resulting in lower simple normative adherence compared to unadjusted adherence. Case B is an example of inhaled therapy under-prescription based on previous exacerbation history resulting in lower sophisticated normative adherence compared to unadjusted adherence and simple normative adherence. Case C is an example of nebulizer overuse exaggerating the magnitude of unadjusted adherence.

**Conclusion:**

Different methods of reporting adherence can result in different magnitudes of adherence. We have proposed two methods of standardizing the calculation of adherence which should better reflect treatment effectiveness. The value of these indices can be tested empirically in clinical trials in which there is careful definition of treatment regimens related to key patient characteristics, alongside accurate measurement of health outcomes.

## Introduction

Cystic fibrosis (CF) is a multisystem genetic condition due to CF transmembrane conductance regulator protein dysfunction resulting in abnormal ion transport across epithelial cells.^1^ It is a progressive and life-limiting condition, characterized by recurrent lower respiratory tract infection leading to lung damage and death from respiratory failure.^1^ Life expectancy has nonetheless been improving with median survival now exceeding 37 years,^2^–^4^ largely due to the increasing availability of effective therapy.^5^ Given the respiratory burden of CF, inhaled medications consisting of antibiotics and mucolytics are the main-stay therapies with multiple randomized controlled trials (RCTs) demonstrating the effectiveness of these treatments.^6^,^7^ However, effective therapy will only work if it is being used appropriately. Various studies have shown that in contrast to adherence rates within RCTs, which typically exceed 80%,^8^,^9^ median medication adherence in clinical practice ranges between 30% and 50%.^10^–^12^ With median adherence below 50% in adults, adherence is an important potential cause of treatment failure,^13^ and medication adherence rates are likely to be an important indicator of the quality of care.

The first step toward using adherence rate as an indicator of the quality of care is to measure it accurately. There is clear evidence that electronic data capture of medication adherence is superior in terms of accuracy compared with self-report or other indirect measures, such as pharmacy refill data.^10^,^11^,^14^ In CF, tamper-proof nebulizer systems which provide date- and time-stamped data for nebulized medication delivered are now available.^15^,^16^ These data logging nebulizers provide rich data that can potentially be used to support adherence^15^ with meta-analysis suggesting that feeding back data to patients can increase adherence by around 20%.^17^ In addition, adherence data are crucial to clinical decision making.^18^ There is little point in responding to increased exacerbations and lung function decline by switching patients from twice daily tobramycin to thrice daily aztreonam lysine if the cause of exacerbations is untreated CF due to nonadherence. Where it is practical to do so, electronic data capture should be integrated into routine care and used as a “gold standard” measure of medication adherence.

Although CF is the most common life-limiting genetic condition in the UK, it is still relatively uncommon with a population of just over 10,000 people in the UK and 70,000 worldwide.^19^ The UK CF registry data from 2014 document that only two out of the 60 UK specialist CF centers (28 adult centers, 32 pediatric centers) have >400 patients.^20^ In a seminal paper in 1995, Mant and Hicks^21^ demonstrated that measuring processes of care proven in RCTs to reduce death could detect meaningful differences in care quality for myocardial infarction with just 75 cases compared to 8,179 cases needed if mortality was used instead as the quality indicator. The relative utility of process and outcome measures in detecting variations in quality of care is particularly relevant to CF. In CF, a small patient population is spread across many hospital units so that outcome measures, such as lung function, will be relatively insensitive in detecting differences in quality of care between units whereas process measures, such as adherence, have the potential to more easily identify important variations in the quality of care.^21^

### Terminology: defining adherence

There is a lack of consensus on the methods to report adherence. Adherence is typically reported as the total number of doses taken as a percentage of the target number of doses agreed between clinicians and patients.^22^ Percentage adherence clearly depends both on the numerator (ie, the actual number of doses taken) and denominator (ie, the target number of doses to be taken). An increase in percentage adherence (which intuitively might be expected to imply more effective treatment) could be due to increase in the number of doses taken, that is, an increase in the numerator (likely to represent an improvement in effectiveness) or a decrease in the target number of doses to be taken each day, that is, a decrease in the denominator (likely to represent a decrease in effectiveness).

In defining adherence, we have adopted and extended the approach used by Horne et al^22^ in the 2005 National Coordinating Centre for the Service Delivery and Organisation report that considers the definitions of compliance, concordance, and adherence as terms to explore patients’ engagement with therapy.

Compliance is defined as “the extent to which the patient’s behavior matches the prescriber’s recommendations.”^22^ This term is used less often nowadays since it is taken to imply a paternalistic lack of collaboration in setting treatment goals.^22^ Concordance is used in various ways and is sometimes used incorrectly as a synonym for adherence.^22^ We use concordance to describe the agreement about the intended treatment regimen that the patient and clinician achieve after a shared discussion. Adherence is the metric that describes the amount of treatment that is taken once the target has been set through the process of discussion that enables the clinician and patient to achieve concordance.^22^ In using the term “adherence” in this way, we acknowledge that the process of reaching concordance is essential to setting the target which will be the denominator of the adherence metric.

A definition of adherence that recognizes the important role of the denominator naturally leads to the need to signpost the decisions made about the denominator when an adherence rate is described. Simply quoting that the patient has an adherence rate of 50% without qualifying the denominator is uninformative. It therefore makes sense to use terminology to describe adherence that gives information about the target treatment regimen (denominator) that has been agreed between the patient and clinician. We thus use the term “normative adherence” to indicate that the target treatment agreed between patient and clinician has taken account of evidence that indicates that the regimen should be effective. Thus, normative regimens lead to an adherence metric where the denominator is chosen on the basis of effectiveness. It should be acknowledged that the evidence that defines effective treatment regimens can be limited and the authority of the normative label can only be as good as the evidence that is available. In proposing the two working definitions of normative adherence (“simple” and “sophisticated” normative adherence), we have proposed definitions that will allow and require empirical testing. The proposition that the adherence definition is normative because it is associated with the outcomes the therapy is reported to achieve can be investigated empirically in data sets where adherence is carefully defined and key outcomes are measured.

When concordance around treatment goals is informed by considerations other than treatment effectiveness (eg, a regimen based on what adults with CF feel they can realistically manage), we must acknowledge that the adherence target has not been driven primarily by evidence of pharmacological effectiveness – we therefore call this adherence “unadjusted”.

Thus, we can see that whereas normative adherence might be used as a process measure linked to outcomes demonstrated in RCTs and therefore have potential as a quality indicator; the unadjusted adherence has less value in this regard. Studies have shown that despite clear guidelines, only around two-thirds of the people with CF were prescribed the maintenance inhaled therapy recommended by guidelines.^23^–^26^ Given this variation in prescribing, simply measuring adherence to a treatment regimen without any assessment of the appropriateness of the regimen gives only a limited indication of the quality of care.

Treatment burden is often cited as a cause for poor adherence among people with CF^27^ and treatment rationalization (eg, dropping inhaled mucolytic to reduce treatment burden) is often employed as a strategy to improve adherence.^28^ Rationalizing treatment would reduce the target number of doses to be taken (ie, the denominator) and inflate unadjusted adherence, yet reduce the effectiveness of a treatment regimen. In this case, an increase in measured adherence does not represent optimal therapeutic effectiveness. Adults with CF who are colonized with *Pseudomonas* will typically be prescribed at least twice daily nebulized antibiotics in addition to once daily nebulized mucolytic.^29^ If that person has been struggling to take even one nebulizer per day, the clinical team may feel that temporarily simplifying the regimen with the aim of taking just one nebulizer per day might help that person build habit and confidence. However, a reduction in the agreed prescription from three nebulizers per day (ie, antibiotics and mucolytic) to just one nebulizer per day (ie, mucolytic only) would increase the unadjusted percentage adherence threefold without necessarily being accompanied by improvement in clinical outcomes, such as reduction in exacerbation frequency or stabilization of lung function.

We therefore propose that medication adherence among people with CF should be reported in a standardized way to allow appropriate interpretation of the adherence data. This approach also has the potential to lay the groundwork for the comparison of specialist CF center performance using the critical process measure of medication adherence.

In this paper, we aim to explore two different indices of inhaled therapy adherence for adults with CF, that is, simple and sophisticated normative adherence, and provide real-life examples of the change in adherence magnitude depending on how adherence is being reported. We focus on inhaled therapy because accurate adherence measurement with electronic data capture is now technically possible and could be made routinely available. In extending our understanding of adherence, we have used patient characteristics that are routinely available in both the UK and US CF registries with the advantage that adherence indicators might be more easily incorporated into these national data sets. In this paper, we have simplified our task to some extent by choosing to concentrate on developing adherence indices that only apply to adults (defined as age 16 years or above) because the normative treatment in CF differs slightly between adults and children.

## Methods to standardize the reporting of medication adherence

A crucial aspect of standardizing the reporting of inhaled therapy adherence is to fix the minimum denominator at an appropriate value based on the recommended therapy given a person with CF characteristics. In this way, we aim to define the normative treatment regimen by linking the person’s characteristics to consensus guidelines. While there is currently little empirical evidence relating composite regimens to outcomes, trials in which there is careful definition of nebulizer regimens related to key patient characteristics alongside accurate measurement of outcomes, such as exacerbations, will have the potential to provide data linking regimens to outcomes.

### Simple normative adherence: adjusting the denominator according to *Pseudomonas* status

In adults, recommended inhaled therapy regimens will almost always include a mucolytic and then further drug choice will be informed by the person’s *Pseudomonas* status. Dornase alfa is the mucolytic with the strongest evidence base for people with CF.^6^ It should be noted that while comparative effectiveness research is still rare in CF,^30^ the small number of trials comparing dornase alfa against alternative mucolytics (such as hypertonic saline and mannitol) failed to demonstrate the superiority of these alternatives.^31^–^33^ The US CF Foundation recommends long-term dornase alfa for people with CF and at least mild lung disease, defined as predicted forced expiratory volume in 1 second (FEV_1_) of 90% or below,^29^ while the European CF Society recommends the routine long-term use of dornase alfa for everyone with CF aged 6 years and above.^34^ There is some evidence that dornase alfa reduces the frequency of pulmonary exacerbation even among those with normal FEV_1_.^35^–^37^ It is therefore justifiable for every adult with CF to be on long-term inhaled dornase alfa, which is given once daily.

People with CF have accelerated lung function decline once they are chronically colonized with *Pseudomonas*.^38^ There is evidence that the decline can be reduced by long-term inhaled antibiotics.^7^,^39^,^40^ Colistimethate sodium and tobramycin are the two most commonly used antibiotics to suppress chronic *Pseudomonas*, while aztreonam lysine is a new treatment available since 2012.^41^ All the main CF guidelines recommend the use of long-term inhaled antibiotics if a person with CF is chronically colonized with *Pseudomonas*.^29^,^42^

Therefore, the *Pseudomonas* status of an adult with CF provides a basis to determine his/her minimum required treatment. An adult with CF should be on at least one inhaled therapy per day (ie, inhaled dornase alfa). If there is evidence of chronic *Pseudomonas* colonization, then he or she should be on a minimum of three inhaled therapies per day long-term (ie, once daily inhaled dornase alfa and twice daily inhaled antibiotic).

Once this has been defined, further adjustment can be made to take account of intermittent inhaled antibiotic regimens.^43^ For example, inhaled tobramycin and inhaled aztreonam lysine are usually prescribed on a 28-day on/off cycles.^29^ For people with chronic *Pseudomonas* whose regimen consists of only one type of intermittent inhaled antibiotics, their minimum denominator would be 3 during the 28-day “on” period and 1 during the 28-day “off” period.

It should also be noted that in some CF centers, inhaled therapy is discontinued during treatment with intravenous antibiotics. In such centers, periods of intravenous therapy need to be excluded from the adherence calculations to take into account agreed missed doses.

### Intermittent *Pseudomonas*

Further regimen adjustments are needed for those with intermittent *Pseudomonas*. There is strong evidence that early inhaled therapy following the first isolation of *Pseudomonas* can successfully eradicate *Pseudomonas* among people with CF.^44^ Latest evidence suggests the success rates of *Pseudomonas* eradication are similar between children and adults.^45^,^46^ All the main CF guidelines strongly recommend *Pseudomonas* eradication,^34^,^42^,^47^ although there is a lack of consensus regarding the eradication regimen. The US CF Foundation recommends 1 month of twice daily tobramycin as the first-line treatment^47^ while the UK CF Trust recommends 3 months of colistimethate sodium.^42^ Therefore, when persons with CF who had been clear of *Pseudomonas* reacquire *Pseudomonas*, they should be treated with twice daily inhaled antibiotics for 1 or 3 months depending on the antibiotic regimen chosen (ie, a minimum of three inhaled therapies per day for 1 or 3 months depending on the antibiotic regimen during the eradication period, then back to one inhaled therapy).

### How can normative adherence be used to understand quality of care across CF units?

Clinical medicine is inherently complex and in many cases progress can only be made if we do not allow the perfect to be the enemy of the good. Improvement in quality of care is supported by measurement that allows feedback to prompt improvement and subsequent reassessment.^48^ A CF unit with normative adherence of 30% might well learn from a unit with normative adherence of 60%, but benchmarking using normative adherence will require units to have confidence that apples are being compared to apples and not to oranges. Whereas understanding adherence data for patients with intermittent *Pseudomonas* is inherently complex, there is broad consensus around treatment for chronic *Pseudomonas*. The 2014 UK CF registry report suggests that nearly 50% of adults with CF are chronically colonized with *Pseudomonas*^20^ and normative adherence for this group will allow important comparisons on a significant proportion of the adults with CF in a specialist CF center.

### Initial steps toward a longer term goal

It is important to note that the initial emphasis in understanding normative adherence among people with CF, such as those with chronic *Pseudomonas*, is to define a minimum denominator value that will allow broad comparison across relatively homogenous groups of people, rather than mandating a denominator that will apply to every person with CF. This is an approach in which the perfect is not allowed to be the enemy of the good and in which starting to measure adherence in a standardized way provides the first step in a longer journey of beginning to understand how much treatment is adequate for an individual with CF, depending on the severity of his/her lung disease. We recognize there is a wide range of lung disease severity and some people would require more than the minimum amount of treatment. For example, someone with chronic *Pseudomonas* might require (and agree to) regular twice daily colistimethate sodium, once daily dornase alfa, and twice daily hypertonic saline solution. In such an individual, the denominator should be 5 instead of 3. The data to achieve this level of precision in the personalization of adherence targets are not yet available. However, the eventual goal in understanding normative adherence should be to define the metric that identifies the level of treatment that will maintain stability and prevent exacerbations given an individual’s characteristics.

While dornase alfa and nebulized antibiotics are considered as the “core treatments” within CF, we recognize that other nebulized treatments, such as hypertonic saline and bronchodilators, are also used by people with CF. There may also be people with CF who just used hypertonic saline ± nebulized bronchodilators. Based on the definition of simple normative adherence whereby everyone should be on at least dornase alfa, this group of people will have an adherence of 0% regardless of the number of hypertonic saline or nebulized bronchodilators used. Although this may seem unsatisfactory, defining the minimum treatment denominator based only on the core treatments is a pragmatic decision to allow consideration of treatment regimen effectiveness.

Another issue would be the occasional (as needed) use of additional doses of hypertonic saline (or nebulized bronchodilator) over and above the core regimen of dornase alfa and inhaled antibiotics. In that case, a pragmatic decision can be made to decide that the core denominator remains at three doses per day and accept that it is impossible to be certain whether the three doses taken are the most important “normative” therapies. If there is regular use of hypertonic saline (or nebulized bronchodilator), then these therapies simply need to be included in the denominator as discussed earlier.

### Unadjusted adherence as a potential quality indicator

It is important to recognize that center comparisons may sometimes need to use data that are not as complete as we would wish it to be. Some CF centers that struggle to deliver high quality care will also struggle to provide data sets with high levels of data completeness. This may well mean that a data capture system that uses chipped nebulizers may automatically provide data on how many total doses of nebulized therapy have been delivered, but the data that require unit level input, such as detailed prescription data or patient characteristics, may be missing.

It will be important to explore the potential of crude unadjusted adherence metrics to identify differences between centers since for some centers the data to allow normative adherence to be calculated may be missing. There are observational data from the US that relatively crude metrics can indicate differences in quality of care.^49^ The ESCF study showed that the centers with the best outcomes reviewed their patients more frequently than those that did not, and this analysis was only stratified according to FEV_1_.^49^ Similarly, centers that used more intravenous antibiotics had better outcomes than those that used less. It may be that an unsophisticated comparison of unadjusted adherence that simply looks at the mean number of nebulized doses taken per patient per year may identify centers delivering different levels of quality of care. Such a metric will consider both the core treatments, such as dornase alfa, and also “add on treatments”, such as nebulized bronchodilators.

It is possible that data linkage between UK CF registry and automatic nebulizer download may allow some coarse adjustment, for example, for FEV_1_ as an indicator of lung disease severity. Although developing high quality metrics adjusted for known confounders is likely to develop the most discriminating and informative comparisons, more work is needed to understand what information can be gleaned from the data sets with missing data.

### Sophisticated normative adherence: adjusting the denominator based on several prognostic factors

Sophisticated normative adherence is an exploratory approach that goes beyond the simple normative adherence by also taking into account exacerbation history from the previous 1 year (in the form of intravenous antibiotics used) to determine the need for inhaled antibiotics. This approach mirrors a published National Health Service (NHS) England guideline informed by the evidence from RCTs.^50^ The NHS England Clinical Commissioning Policy recommends that it would be appropriate to escalate to thrice daily aztreonam lysine for people with CF who are having more than two episodes of exacerbations annually or losing >2% of FEV_1_ per year despite alternating regimen of nebulized tobramycin and colistimethate sodium.^50^ Hence, exacerbation frequency is already being used to personalize normative adherence within the NHS England commissioning guidelines.

An understanding of the importance of exacerbation frequency in determining the choice of inhaled antibiotics may provide a helpful starting point in deciding treatment regimens for people with CF in whom *Pseudomonas* status is difficult to define. Although there are clear guidelines for diagnosing “chronic *Pseudomonas*” in people with CF,^51^,^52^ there are occasions whereby the diagnosis is less straightforward. Not everyone with CF is able to expectorate sputum, and cough swabs have limited sensitivity in detecting *Pseudomonas*.^53^–^55^ Although anti-*Pseudomonas* antibody is useful in some cases, not every CF center has access to good quality and rapid anti-*Pseudomonas* antibody results.^51^ Intermediate values of anti-*Pseudomonas* antibody are also difficult to interpret.^56^ The Leeds definition (which does not rely on anti-*Pseudomonas* antibody levels^51^) remains the most commonly used method to determine the *Pseudomonas* status of a person with CF. However, with 4,818 of the 39,326 participants in the observational ESCF study with indeterminate *Pseudomonas* status based on the Leeds definition,^57^ determining the requirement for inhaled antibiotics based on *Pseudomonas* status alone may be inadequate. There are people without *Pseudomonas* infection who still have frequent exacerbations,^58^,^59^ and there is a wide range of exacerbation frequency even among those with chronic *Pseudomonas*.^60^ A history of more than one previous exacerbation in the previous year is a strong risk factor for more frequent exacerbations.^60^–^62^ It could be argued that people with CF experiencing more frequent exacerbations and having higher intravenous antibiotics requirement would benefit from inhaled antibiotics. Thus, in people without confirmed evidence of chronic *Pseudomonas* who might otherwise simply be on an inhaled mucolytic, it could make sense to use a history of more than one previous exacerbation in the previous year as a reason to recommend the need for inhaled antibiotic therapy. Once adherence measurement is embedded within CF registries, the benefit of this approach can be investigated empirically.

The requirement for intravenous antibiotics has been used as a pragmatic marker for the frequency and severity of pulmonary exacerbations in various clinical trials and observational studies.^63^–^69^ Higher requirement for intravenous antibiotics is an independent predictor for higher mortality.^63^,^64^ Large observational studies showed a median number of exacerbations per year of around one among adults with CF,^57^,^58^ which corresponds with around 14 days of intravenous antibiotics.^70^ Therefore, a cutoff of 14 days in the past year is clinically reasonable to differentiate between those with “low” and “high” intravenous antibiotics requirement. According to the sophisticated normative adherence index, someone with >14 days of intravenous antibiotics use in the previous 1 year should be on once daily inhaled dornase alfa and twice daily inhaled antibiotic for the current year regardless of his or her *Pseudomonas* status.

### Numerator adjustments

In defining simple and sophisticated normative adherence, we have attempted to produce a definition of adherence that might be expected to ensure that a higher percentage adherence to the specified regimen is associated with greater effectiveness (in terms of controlling inflammation, limiting exacerbations, and minimizing FEV_1_ decline) and a lower percentage adherence associated with lower effectiveness. These normative adherence indices achieve this by adjusting the denominator (the drugs within the regimen) in the light of a person with CF’s clinical characteristics. However, just as the drugs within a regimen (the denominator) might be expected to influence effectiveness, the way in which the drugs are taken (the numerator) will also be important. Unless the influences of the numerator are understood, a measure of normative adherence may not accurately estimate regimen effectiveness.

The most important numerator adjustment is to cap the daily maximum inhaled therapy use at 100%. While “medication dumping” is difficult with I-neb^®^ (Philips Respironics, MA, USA), which only emits aerosol on inspiration, overuse of medication (ie, adherence >100%) at a certain period can inflate the unadjusted adherence figures without necessarily improving health outcomes. For example, after missing inhaled therapy over the weekend, a person may try to compensate by using more than the recommended doses of medication on Monday. Such a strategy might allow untreated inflammation to produce lung damage during “treatment holidays”. If this possibility is not accommodated within the definition of adherence that explores the impact of adherence on health outcomes, the magnitude of adherence may fail to detect the impact of inconsistent use.

By imposing a limit of 100% adherence per day (ie, excess nebulizer use is discounted), the calculated adherence magnitude will better reflect the effective doses of medication used.

When calculating normative adherence by combining numerator and denominator adjustments, it is important to do the numerator adjustment first (so that maximum number of nebulizers used per day is no >100%) prior to dividing the number of nebulizers used by the appropriate normative denominator. It is also important to note that the maximum number of nebulizers used per day is limited by the agreed prescription instead of the normative denominator. For example, if someone is only prescribed colistimethate sodium but needs colistimethate sodium and dornase alfa due to chronic *Pseudomonas*, even if the person used three colistimethate sodium doses per day, his/her maximum daily nebulizer use is still limited at two because the maximum recommended colistimethate sodium use is twice daily and the prescribed treatments do not include dornase alfa (which means that person does not possess the medications required to achieve 100% normative adherence).

The sophisticated normative adherence index can be further extended to take into account other technical factors that may influence treatment effectiveness, such as the need to use the I-neb twice to receive a full dose of nebulized tobramycin. These issues are discussed in the Supplementary materials.

## Practical examples of adherence reported with the normative adherence indices

We present three composite cases which contain typical characteristics of people with CF based on our experience to provide practical examples of calculating the normative adherence indices. These cases are summarized in Table 1. These are illustrative rather than real cases.

### Illustrative case A: under-prescription of inhaled therapy as defined by *Pseudomonas* status

Person A is in his mid-30s and has chronic *Pseudomonas* in 2014 based on the Leeds definition.^51^ His best FEV_1_ in 2014 was 116%. He did not require any intravenous antibiotics in 2013.

He was prescribed regular twice daily nebulized colistimethate sodium only. His dornase alfa was discontinued at his request in July 2011 because he struggled to use his inhaled therapy.

He used 203 nebulizers throughout 2014. His agreed prescription was twice daily nebulized colistimethate sodium, so his agreed target was 730 doses. Therefore, his unadjusted nebulizer adherence for 2014 was 203/(2×365) =27.8% of the agreed or prescribed doses.

According to the simple normative adherence index, his chronic *Pseudomonas* meant that his minimum inhaled therapy would be once daily inhaled mucolytic and twice daily inhaled antibiotic throughout 2014. Therefore, the denominator for calculating his adherence should have been 3 throughout 2014 (instead of 2). His simple normative adherence index for 2014 would therefore be 203/(3×365) =18.5%.

His sophisticated normative adherence index for 2014 is identical to his simple normative adherence because his *Pseudomonas* status means that he should be on both inhaled antibiotics and a mucolytic.

Numerator adjustment did not affect his normative adherence index because there was no inappropriate nebulizer use or nebulizer overuse. When calculating the normative adherence with both numerator and denominator adjustments, it is important to do the numerator adjustment first to determine his maximum daily nebulizer use prior to dividing that figure with the normative denominator (which was 3 in this case due to chronic *Pseudomonas*). Although his normative denominator was 3, his maximum daily nebulizer use was limited at 2 because the maximum recommended colistimethate sodium use is twice daily. Person A did not use more than two nebulizers in a single day in 2014; hence, even with the numerator adjustment capping his daily maximum nebulizer use per day at two, his total normative numerator for 2014 remained at 203 doses. Therefore, his simple normative adherence with numerator adjustment for 2014 would still be 203/(3×365) =18.5%.

### Illustrative case B: under-prescription of inhaled therapy based on intravenous antibiotic requirement

Person B is in her early 30s who has not cultured *Pseudomonas* since 2011, but was mainly providing cough swabs. Her best FEV_1_ in 2014 was 64%. She required 42 days of intravenous antibiotic in 2013.

She was on dornase alfa once daily throughout 2014, whereby she used 223 nebulizers out of the 365 agreed doses. Therefore, her unadjusted nebulizer adherence for 2014 was 61.1% of the agreed or prescribed doses. Her simple normative adherence without numerator adjustment for 2014 was also 61.1% because no denominator adjustment was needed given that she did not culture *Pseudomonas* in 2014.

Sophisticated normative adherence uses exacerbation frequency data as an indicator of untreated inflammation/infection and escalates recommended treatment to respond to this. Based on her intravenous antibiotics requirement being >14 days in 2013, her sophisticated normative adherence would indicate that she required inhaled antibiotic throughout 2014. Her sophisticated normative adherence without numerator adjustment for 2014 would therefore be 20.4%, based on a denominator of 3 per day (instead of 1 per day).

Her normative adherence indices for 2014 remained identical following numerator adjustment (61.1% for simple normative adherence, 20.4% for sophisticated normative adherence) because there was no inappropriate nebulizer use or nebulizer overuse.

The difference between simple and sophisticated normative adherence is marked in this case and it should be noted that sophisticated normative adherence is an exploratory concept that will require empirical testing in clinical practice.

### Illustrative case C: brief periods of nebulizer overuse resulted in a slightly exaggerated unadjusted adherence

Person C is in his mid-30s with chronic *Pseudomonas* in 2014 based on the Leeds definition.^51^ His best FEV_1_ in 2014 was 85% and he required 38 days of intravenous antibiotics in 2013.

Throughout 2014, he was on once daily dornase alfa and twice daily nebulized colistimethate sodium alternating every 2 weeks with twice daily nebulized tobramycin. It is important to note that he was taking his tobramycin through the I-neb, which requires two separate nebulizations to take a single full dose, so twice daily tobramycin requires four nebulizations.

He completed 848 nebulizer treatments throughout 2014. Of note, from October to November 2014, he was using dornase alfa twice daily.

Given that he was already prescribed both inhaled mucolytic and antibiotic (ie, no adjustments to the denominator are required to calculate his normative adherence), his unadjusted adherence and normative adherence without numerator adjustment (for both simple and sophisticated) were all identical at 64.5%.

However, with numerator adjustment, both his normative adherence indices would be slightly lower at 58.2% due to the capping of daily maximum adherence at 100%, which negates the effect of excess dornase alfa use. Following numerator adjustment limiting his maximum daily nebulizer use at 3 per day (in accordance with his agreed prescription of once daily dornase alfa and twice daily nebulized antibiotics), he only used 637 doses of effective nebulized treatments in 2014. Therefore, his normative adherence with both denominator and numerator adjustments would be 637/(3×365) =58.2%.

## Discussion

We have described the principles of adjusting adherence data to standardize the reporting of adherence rates using an approach that links reported adherence rates to evidence on effectiveness. The aim is to make adherence rates easier to interpret and allow comparison of quality of care. The most important aspect of simple normative adherence is to set a minimum value for treatment, which defines the denominator of the adherence rate based on the inhaled therapy targets for a given person with CF. These targets are derived from consensus guidelines informed by RCTs.^34^,^42^,^47^ Adjusting the numerator potentially adds further useful information and provides a more accurate reflection of how inhaled therapy is being used by people with CF by capping the daily maximum adherence at 100%. We have also provided practical examples of the common scenarios, whereby adherence magnitude may change depending on how it is reported.

The term normative was first used in the context of medication adherence in the National Coordinating Centre for the Service Delivery and Organisation report by Horne et al.^22^ In this report, normative was used to represent “good” and “right” medication taking. We used the term normative adherence to describe adjusted adherence indices based on the right regimen, whereby the right medication regimen is the regimen that the evidence suggests will be effective in delivering the outcomes that the medication is prescribed to achieve according to the RCT evidence. Typical outcomes the medication is “advertised” to deliver will be reduction in pulmonary exacerbations or improvement in lung function. Hence, normative adherence indices are more than just adherence (described as the extent to which a patient’s behavior matches an agreed treatment plan)^22^ in that normative adherence takes into account whether the agreed treatment is likely to be the optimum regimen for a person with CF given his/her characteristics. In this way, the normative adherence rate evaluates the prescribing practices of health care professionals as well as medication use by people with CF.

The approach we have taken in CF is similar to the approach of the Global Initiative for Chronic Obstructive Lung Disease guideline in recommending COPD treatments.^71^ While we have started with the CF clinical guidelines that recommend treatments on the basis of some patient characteristics,^29^,^34^,^42^ in developing the sophisticated normative adherence index, we have extended the patient characterization to include a proxy for exacerbations that is available for all UK and US patients in the form of annual intravenous days within the national CF registries. This paves the way for quality assessment across registry data using the well-characterized process measure of normative adherence.

We have presented two different methods of calculating normative adherence. Accurate medication adherence measures should predict health outcomes.^72^ We think it is likely that adjusting the denominator according to a person’s *Pseudomonas* status ± history of exacerbation in the previous 1 year along with capping the daily maximum inhaled therapy use at 100% should improve the accuracy of an adherence index in predicting health outcomes. The relationship between normative adherence and health outcomes, such as intravenous days, should be testable within registry data once adherence measures become embedded in clinical care. The technology to allow this is available and starting to become increasingly used.^18^ An appropriate data set to compare the predictive values of different normative adherence definitions would be an adequately powered RCT of adherence intervention among people with CF. Such a trial has been funded by the National Institute of Health Research and the pilot phase will begin in May 2016.^73^

There are additional potential adjustments to both the numerator and denominator that could be made to further improve the accuracy of the normative adherence index, and these are discussed in the Supplementary materials.

Normative adherence can be viewed as another dimension of personalized medicine.^74^ Developing a detailed understanding of an individual’s normative adherence holds out the promise of defining the minimum amount of adherence required for maintaining optimal lung health. Given the increasing treatment options in CF,^75^ understanding “how much adherence is enough” will be vital in helping to tackle the increasing CF treatment complexity.^76^

## Supplementary materials

### Further considerations in calculating the “sophisticated” normative adherence index

#### Extending sophisticated normative adherence: further considerations for the denominator adjustment

The precision of the “sophisticated” adherence index might be increased by also taking into account lung function and the severity of the underlying cystic fibrosis (CF) by considering genotype and pancreatic status. For convenience, we will call this index “extended sophisticated” normative adherence. Whether the additional complexity is of value can potentially be tested empirically and care has been taken to only select adjustment factors that are available in the national CF registries to make such empirical testing feasible in the future.

The additional factors involved in the extended sophisticated normative adherence would allow the index to be sufficiently discriminating to provide guidance as to whether a given adult with CF would require dornase alfa rather than simply assuming that all adults with CF should be prescribed dornase alfa. This has particular relevance because widespread genetic testing is identifying rarer cystic fibrosis transmembrane conductance regulator (CFTR) mutations, leading to CF centers providing care to an increasing population of older and “atypical” cases that would otherwise not be diagnosed as CF.^1^ A small group of people with CF have very mild clinical manifestations and near normal lung function even at an sophisticated age.^2^–^4^ In this group of people, there is likely to be less of a consensus about the blanket use of inhaled mucolytic.

Thus, the extended sophisticated normative adherence would potentially identify a group of people who need not necessarily be on any inhaled therapy based on no evidence of *Pseudomonas*, no history of frequent exacerbations (with the requirement of >14 days of intravenous antibiotics in 1 year as a marker of frequent exacerbation), forced expiratory volume in 1 second (FEV_1_) >90%, pancreatic sufficient, and “mild genotype” (at least one class IV–V CFTR mutation^5^).

FEV_1_ >90% is accepted by the US CF Foundation as “normal lung function”, whereby long-term dornase alfa is not considered essential.^6^ Pancreatic insufficiency is an independent risk factor for increased FEV_1_ decline among people with CF^7^–^9^ and is associated with poorer prognosis.^1^ A potential disadvantage of relying solely on the pancreatic status to identify milder phenotype is that people with mild phenotype who were initially pancreatic sufficient may eventually become pancreatic insufficient after a series of episodes of pancreatitis.^10^ Although there is significant phenotypic variability for each class of CFTR mutation, the relationship between pancreatic status and genotype is more robust and the group with at least one class IV–V CFTR mutation does tend to have milder lung disease.^2^,^5^,^11^,^12^ Therefore, the genotype is useful in supplementing the information provided by pancreatic status in terms of confirming that an individual has a milder phenotype.

Figure S1 summarizes the rubric for combining the different prognostic factors used to determine the required maintenance inhaled therapy for this form of sophisticated normative adherence index.

#### Taking into account incomplete doses: numerator adjustment for adherence levels calculated from I-neb^®^ data

The I-neb^®^ records four different readings for each nebulizer dose depending on treatment completeness: “full” = full nebulizer dose delivered; “12.5%–100%” = treatment taken but incomplete dose; “<12.5%” = treatment attempted but unlikely to receive any; and “none” = I-neb^®^ switched on but no treatment taken.^13^ To ensure that the number of treatments is correctly calculated, a ”full” dose is counted as “1 dose”, “12.5%–100%” is counted as “½ dose” while “<12.5%” and “none” doses are counted as 0.

#### Taking into account doses taken after midnight: numerator adjustment for irregular lifestyles

When a limit of 100% adherence per day is being used as part of numerator adjustment, it can be informative to recognize that many young people will go to bed after midnight. It is not uncommon for these “night owls” to use their inhaled therapy after midnight (eg, after returning from a night out). For example, a person may use his inhaled antibiotic at 10 am and take the final dose of the day just before bed, which may on occasions be 1 or 2 am. Let us say the second lot of inhaled therapy (this time an inhaled antibiotic and inhaled dornase alfa) was used around 1 am the next morning. He/she woke up around 10 am to start his/her new day and used his/her morning inhaled antibiotic. Finally, he/she used his/her second lot of inhaled therapy (inhaled antibiotic and inhaled dornase alfa) at around 11 pm. The unadjusted adherence over the 2 days would be 100% (six nebulizers used out of six prescribed). Capping the daily maximum at 100% using a rigid midnight-to-midnight day would result in one nebulizer counted for the first day and three nebulizers counted for the second day (ie, adherence over the 2 days would only be 67%). Counting a day as starting at 5 am and ending at 4.59 am is a pragmatic solution to this problem, since it is likely that on most occasions a person would go to bed by 5 am and would wake up for the day by 4 pm.^14^

Therefore, to prevent intermittent late doses pushing some days over 100% and leaving other days under 100%, which would lead to a lower overall adherence level due to capping the maximum daily inhaled therapy use at 100%, “a day” should be considered to start at 5 am.

#### Taking into account dose spacing: numerator adjustment for inhaled antibiotics

Another factor that could be considered in ensuring that the adherence index most accurately reflects medication effectiveness is to consider dose spacing.

The common inhaled antibiotic therapies (colistimethate sodium and tobramycin) in CF should be used twice daily, that is, every 12 hours, while inhaled aztreonam lysine dosing is thrice daily. Inhaled antibiotic doses used too close together may not be as beneficial as doses used at the recommended intervals. The UK CF Trust recommends a minimum interval of at least 6 hours for inhaled colistimethate sodium and tobramycin.^15^ Therefore, the numerator adjustment could exclude the inhaled antibiotic doses that were used <6 hours after an initial dose.

#### Taking into account device dose delivery characteristics: numerator adjustment for nebulized tobramycin via I-neb^®^

Nebulized tobramycin via the I-neb requires two separate nebulizations to complete a single dose due to the size of the chamber. Thus, the number of nebulizations per day will differ depending on whether the patient is on a mucolytic and tobramycin or mucolytic and colistimethate sodium. A person would take one nebulization for the dornase alfa and one nebulization for the colistimethate sodium in the morning and just one nebulization for the colistimethate sodium in the evening with a target of three nebulizations per day. If the patient was taking dornase alfa and tobramycin via the I-neb^®^, the morning nebulization target would be one nebulization for dornase alfa and the patient would need to use the nebulizer twice to deliver the full dose of tobramycin. Hence, the patient would have a target of five nebulizations per day.

A relatively common scenario is for people to use both an inhaled mucolytic and inhaled antibiotic within a “treatment session”, but miss their other inhaled antibiotic for the day. If the inhaled antibiotic is colistimethate sodium, the adherence would be 67% (two out of three nebulizers used). However, if the inhaled antibiotic is tobramycin solution, which requires two separate nebulizations for a complete dose via the I-neb, the adherence would only be 60% (three out of five nebulizers used). This discrepancy does not arise with other types of nebulizers, for example, the eFlow Rapid^®^, which does not require the tobramycin solution to be nebulized twice for a complete dose. Missing a dose of colistimethate sodium via the I-neb^®^ should carry the same weight as missing a dose of tobramycin. Therefore, the numerator adjustment counts each nebulization of tobramycin solution via the I-neb^®^ as “½ dose”, so that the complete dose (two nebulizations) would count as “1”. This allows the daily denominator to stay at “3” for those on both inhaled antibiotic and inhaled dornase alfa, thus avoiding the discrepancy between missing a dose of colistimethate sodium versus missing a dose of tobramycin.

Figure S1 The required maintenance inhaled therapy based on a range of prognostic factors used to decide the minimum denominator for the “extended sophisticated” normative adherence.
**Notes:** ^ψ^*Pseudomonas* status as defined by the Leeds definition.^16^ People with intermittent *Pseudomonas* should be on inhaled antibiotics in addition to inhaled mucolytic for 1 month or 3 months depending on the antibiotic regime when *Pseudomonas* is newly detected. ^Ω^Genotype status as defined by international consensus.^5^ “Mild genotype” is defined by the presence of at least one class IV–V CFTR mutation.
**Abbreviations:** CFTR, cystic fibrosis transmembrane conductance regulator; FEV_1_, forced expiratory volume in 1 second; IV, intravenous.

References1HooZHWildmanMJTeareMDExploration of the impact of ‘mild phenotypes’ on median age at death in the U.K. CF registryRespir Med201410857167212467523810.1016/j.rmed.2014.02.0122McKoneEFEmersonSSEdwardsKLAitkenMLEffect of genotype on phenotype and mortality in cystic fibrosis: a retrospective cohort studyLancet20033619370167116761276773110.1016/S0140-6736(03)13368-53RodmanDMPolisJMHeltsheSLLate diagnosis defines a unique population of long-term survivors of cystic fibrosisAm J Respir Crit Care Med200517166216261559147410.1164/rccm.200403-404OC4SimmondsNJCullinanPHodsonMEGrowing old with cystic fibrosis – the characteristics of long-term survivors of cystic fibrosisRespir Med200910346296351902264310.1016/j.rmed.2008.10.0115CastellaniCCuppensHMacekMJrConsensus on the use and interpretation of cystic fibrosis mutation analysis in clinical practiceJ Cyst Fibros2008731791961845657810.1016/j.jcf.2008.03.009PMC28109546MogayzelPJJrNaureckasETRobinsonKACystic fibrosis pulmonary guidelines. Chronic medications for maintenance of lung healthAm J Respir Crit Care Med201318776806892354087810.1164/rccm.201207-1160oe7KonstanMWMorganWJButlerSMRisk factors for rate of decline in forced expiratory volume in one second in children and adolescents with cystic fibrosisJ Pediatr200715121341391764376210.1016/j.jpeds.2007.03.0068KeremEVivianiLZolinAFactors associated with FEV1 decline in cystic fibrosis: analysis of the ECFS patient registryEur Respir J20144311251332359895210.1183/09031936.001664129KonstanMWWagenerJSVanDevanterDRRisk factors for rate of decline in FEV1 in adults with cystic fibrosisJ Cyst Fibros20121154054112256136910.1016/j.jcf.2012.03.009PMC408618910AugartenABen TovAMadgarIThe changing face of the exocrine pancreas in cystic fibrosis: the correlation between pancreatic status, pancreatitis and cystic fibrosis genotypeEur J Gastroenterol Hepatol20082031641681830129410.1097/MEG.0b013e3282f36d0411No authors listedCorrelation between genotype and phenotype in patients with cystic fibrosis. The Cystic Fibrosis Genotype-Phenotype ConsortiumN Engl J Med19933291813081313816679510.1056/NEJM19931028329180412McKoneEFGossCHAitkenMLCFTR genotype as a predictor of prognosis in cystic fibrosisChest20061305144114471709902210.1378/chest.130.5.144113NikanderKArhedenLDenyerJCobosNParents’ adherence with nebulizer treatment of their children when using an adaptive aerosol delivery (AAD) systemJ Aerosol Med20031632732811457232510.1089/08942680376901764014HooZHGardnerBCurleyRWildmanMJPart III: constructing a process behaviour (XmR) chart with I-neb adherence data for people with cystic fibrosisJ Im Sci20131211215UK Cystic Fibrosis Trust Antibiotic Working GroupAntibiotic treatment for cystic fibrosis52009[cited January 31, 2016]. Available from: https://www.cysticfibrosis.org.uk/~/media/documents/the-work-we-do/care/consensus-documents/antibiotic-treatment-cystic-fibrosis-may-09.ashx?la=enAccessed January 31, 201616LeeTWBrownleeKGConwaySPDentonMLittlewoodJMEvaluation of a new definition for chronic Pseudomonas aeruginosa infection in cystic fibrosis patientsJ Cyst Fibros20032129341546384310.1016/S1569-1993(02)00141-8

## Figures and Tables

**Table 1:** Summary of the different adherence values depending on how the medication adherence is reported

Example	Unadjusted adherence (%)	“Simple” normative adherence (without numerator adjustment) (%)	“Sophisticated” normative adherence (without numerator adjustment) (%)	“Simple” normative adherence with numerator adjustment (%)	“Sophisticated” normative adherence with numerator adjustment (%)	Reasons for discrepancy
Person A	27.8	18.5	18.5	18.5	18.5	Under-prescription of inhaled therapy based on *Pseudomonas* status
Person B	61.1	61.1	20.4	61.1	20.4	Under-prescription of inhaled therapy based on previous intravenous antibiotic requirement
Person C	64.5	64.5	64.5	58.2	58.2	Brief period of nebulizer overuse resulted in slightly exaggerated unadjusted adherence figure
