# Supplementary figures and images for: Accurate reporting of adherence to inhaled therapies in adults with cystic fibrosis: methods to calculate “normative adherence”
Source: Patient Prefer Adherence. 2016 May 23;10:887–900. doi: 10.2147/PPA.S105530 (PMC4883819; doi:10.2147/PPA.S105530)

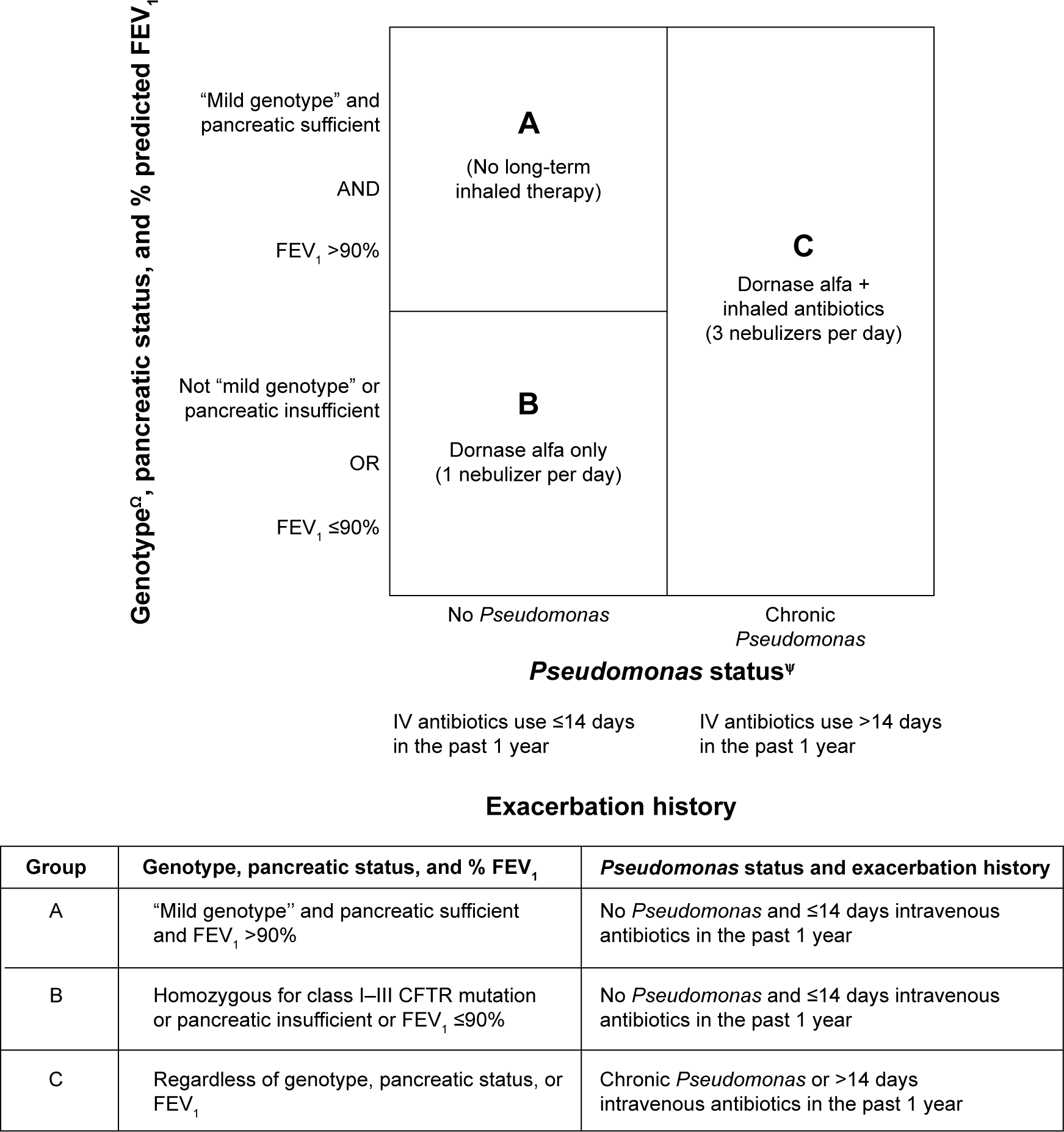

Supplement: Figure S1 — The required maintenance inhaled therapy based on a range of prognostic factors used to decide the minimum denominator for the “extended sophisticated” normative adherence. Notes: ψPseudomonas status as defined by the Leeds definition.16 People with intermittent Pseudomonas should be on inhaled antibiotics in addition to inhaled mucolytic for 1 month or 3 months depending on the antibiotic regime when Pseudomonas is newly detected. ΩGenotype status as defined by international consensus.5 “Mild genotype” is defined by the presence of at least one class IV–V CFTR mutation. Abbreviations: CFTR, cystic fibrosis transmembrane conductance regulator; FEV1, forced expiratory volume in 1 second; IV, intravenous. [file ppa-10-887s1.tif]
